# Dysregulation of tryptophan catabolism at the host-skin microbiota interface in hidradenitis suppurativa

**DOI:** 10.1172/jci.insight.140598

**Published:** 2020-10-15

**Authors:** Laure Guenin-Macé, Jean-David Morel, Jean-Marc Doisne, Angèle Schiavo, Lysiane Boulet, Véronique Mayau, Pedro Goncalves, Sabine Duchatelet, Alain Hovnanian, Vincent Bondet, Darragh Duffy, Marie-Noëlle Ungeheuer, Maïa Delage, Aude Nassif, James P. Di Santo, Caroline Demangel

**Affiliations:** 1 Immunobiology of Infection Unit, Institut Pasteur, INSERM U1221, Paris, France.; 2 Université Paris 7, Sorbonne Paris Cité, Paris, France.; 3 ENS de Lyon, Lyon, France.; 4 Innate Immunity Unit, Institut Pasteur, INSERM U1223, Paris, France.; 5 Laboratoire de Biochimie Hormonale et Nutritionnelle, CHU Grenoble-Alpes, La Tronche, France.; 6 Université de Paris, Imagine Institute, Laboratory of Genetic Skin Diseases, INSERM UMR 1163, Paris, France.; 7 Department of Genetics, Assistance Publique–Hôpitaux de Paris, Hôpital Necker-Enfants Malades, Paris, France.; 8 Immunobiology of Dendritic Cells, Institut Pasteur, INSERM U1223, Paris, France.; 9 ICAReB platform and; 10 Centre Médical, Institut Pasteur, Paris, France.

**Keywords:** Dermatology, Inflammation, Amino acid metabolism, Cellular immune response, Skin

## Abstract

Hidradenitis suppurativa (HS) is a chronic skin disorder of unknown etiology that manifests as recurrent, painful lesions. Cutaneous dysbiosis and unresolved inflammation are hallmarks of active HS, but their origin and interplay remain unclear. Our metabolomic profiling of HS skin revealed an abnormal induction of the kynurenine pathway of tryptophan catabolism in dermal fibroblasts, correlating with the release of kynurenine pathway–inducing cytokines by inflammatory cell infiltrates. Notably, overactivation of the kynurenine pathway in lesional skin was associated with local and systemic depletion in tryptophan. Yet the skin microbiota normally degrades host tryptophan into indoles regulating tissue inflammation via engagement of the aryl hydrocarbon receptor (AHR). In HS skin lesions, we detected contextual defects in AHR activation coinciding with impaired production of bacteria-derived AHR agonists and decreased incidence of AHR ligand-producing bacteria in the resident flora. Dysregulation of tryptophan catabolism at the skin-microbiota interface thus provides a mechanism linking the immunological and microbiological features of HS lesions. In addition to revealing metabolic alterations in patients with HS, our study suggests that correcting AHR signaling would help restore immune homeostasis in HS skin.

## Abstract 2

Loss of homeostasis of tryptophan metabolism at the host-microbiota interface may contribute to Hidradenitis Suppurativa.

## Graphical abstract

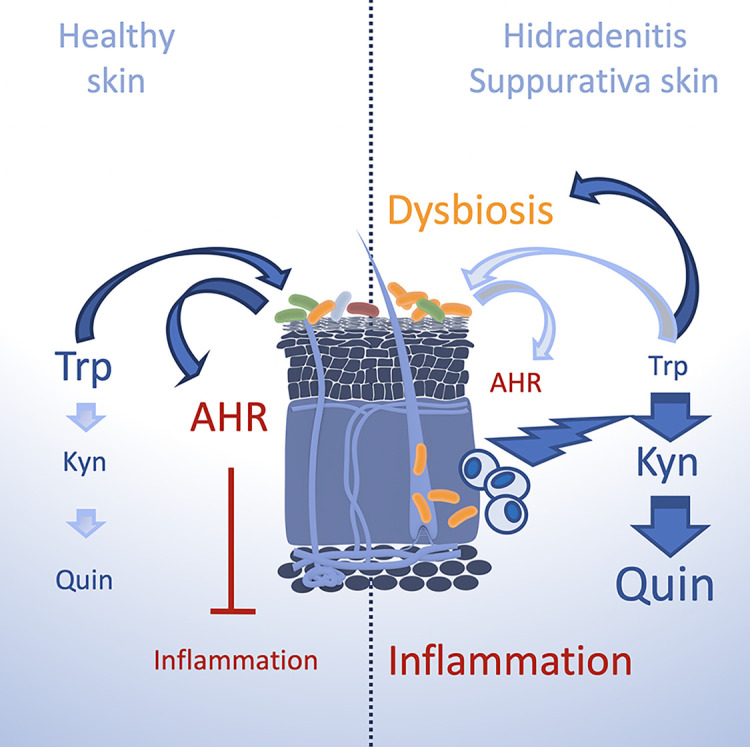

## Introduction

Hidradenitis suppurativa (HS), also known as Verneuil’s disease and acne inversa, is an inflammatory disease of the pilosebaceous follicle, which typically starts in the second decade of life. The average prevalence of HS is 1%, with a 3-fold greater incidence in women than men (1). In most patients (68%), HS manifests as recurrent subcutaneous nodules or abscesses (stage I lesions in Hurley’s severity classification). Some patients develop more severe forms of the disease, with fistulas (Hurley II, 28%) or multiple interconnecting lesions (Hurley III, 4%). In addition to causing intense pain, HS is commonly associated with depressive symptoms and anxiety (2). There is no standard treatment or definitive cure for HS, but immunomodulatory drugs, antibiotics, and surgical resection can reduce symptoms (1).

The etiology of HS is poorly understood (3). While mutations in γ-secretase genes were detected in a small subset of patients with HS, their link with the pathophysiology of HS has remained elusive (4). For the large majority of cases, HS is currently believed to involve defects in genes regulating inflammation and immunity (5). In support of this view, persistent production of proinflammatory IL-1β is a distinctive feature of HS lesions (6–8). TNF-α is also frequently induced in advanced HS lesions, and the TNF-α blocker adalimumab had a positive effect on the clinical outcome of moderate-to-severe HS (9). Infiltrating myeloid cells are typically found in skin lesions, with antimicrobial peptide and IFN signatures reflecting the generation of potent innate immune responses to infection (6, 10–15). An aberrant activation of neutrophils and B cells was also reported, which may jointly contribute to perpetuate lesion inflammation in patients with HS (12, 13). Finally, T cells recruited to HS skin lesions produce IL-17A and IFN-γ but little IL-22 (6, 7, 10, 16–19), an immune signature contrasting with the increased frequency of Th17 and Th22 cells, but not Th1 cells, in peripheral blood (6, 10–13, 19).

In parallel with dysregulated immune responses, metagenomic analyses have identified a distinctive anaerobic microbiota in HS skin, the expansion of which correlates with lesion severity (20, 21). Antibiotic combinations targeting these anaerobic bacteria efficiently eliminated HS symptoms, and remission could be prolonged with monotherapy maintenance treatments (22–26). Interestingly, inflammatory bowel disease and metabolic syndrome are frequent HS comorbidities (27–30). In both of these conditions, unresolved inflammation was linked to perturbations of the gut microbiota-host metabolic homeostasis. We therefore hypothesized that metabolic alterations may link the cutaneous dysbiosis and immune dysregulation characterizing HS pathophysiology. This prompted us to conduct a metabolomic analysis of HS skin.

## Results

### The kynurenine pathway of tryptophan catabolism is activated in HS skin lesions.

**Figure 1:**
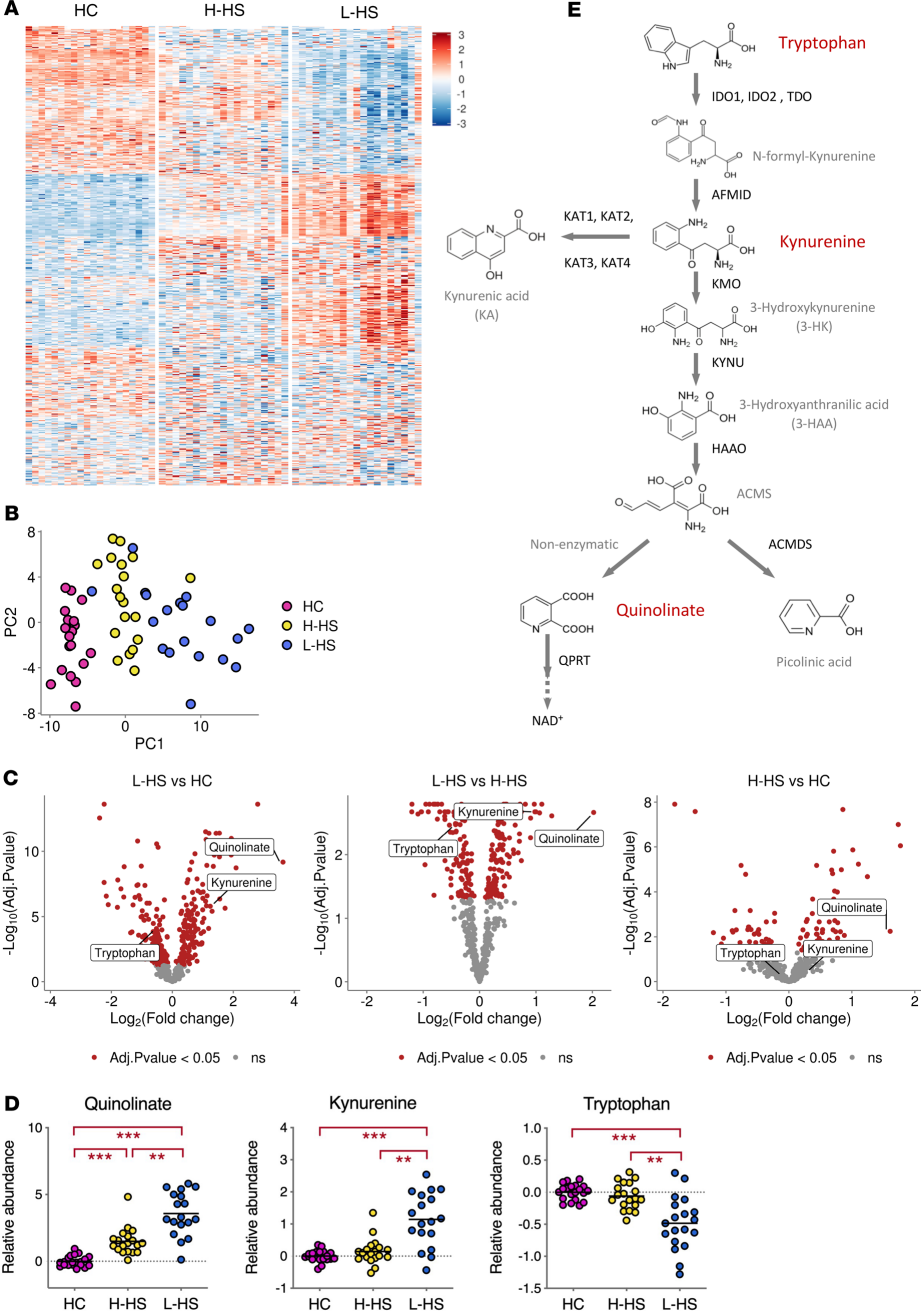
The kynurenine pathway of Trp catabolism is activated in HS skin lesions. (**A**) Heatmap showing the relative abundance of metabolites in skin biopsies from HC and patients with HS (H-HS, healthy skin; L-HS, lesional skin). (**B**) Principal component analysis (PCA) based on metabolite abundance in skin samples. Axes correspond to the 2 first principal components. (**C**) Volcano plots illustrating pairwise comparisons of relative metabolite levels between groups. (**D**) Relative levels of Trp metabolites in skin samples from HC and patients with HS, displayed as scatter dot plots with means. **P < 0.01, ***P < 0.001 by Welch’s 2-sample *t* test for comparisons with HC and matched pairs *t* test for comparisons of L-HS with matched H-HS, with Benjamini-Hochberg correction for multiple comparisons. (**E**) Kynurenine pathway of Trp catabolism.

To capture the biochemical changes coinciding with the most frequent form of the disease, we compared the metabolomes of paired lesional and clinically normal skin samples from patients with Hurley stage I HS (Supplemental Table 1; supplemental material available online with this article; https://doi.org/10.1172/jci.insight.140598DS1). Biopsy specimens were also harvested from healthy subjects in order to detect constitutive alterations in HS skin metabolism. Our semiquantitative analysis detected a total of 500 metabolites, which distributed differently among skin samples from healthy controls (HC) and healthy and lesional skin from patients with HS (H-HS and L-HS, respectively) (Figure 1A). A principal component analysis clearly discriminated the metabolomes of HC and patients with L-HS, while H-HS samples showed an intermediate profile (Figure 1B).

We found that 335 metabolites were differently modulated between patients with L-HS and HC, among which 165 were significantly augmented in patients with L-HS (Supplemental Table 2). Notably, the most upregulated metabolite in patients with L-HS, compared with both HC and patients with H-HS, was quinolinate (Quin) (Figure 1, C and D). Quin levels were also significantly upregulated in patients with H-HS, compared with HC (Figure 1, C and D). Quin is a downstream product of the kynurenine (Kyn) pathway of tryptophan (Trp) catabolism culminating in NAD^+^ production (Figure 1E). Similar to Quin, Kyn was significantly upregulated in patients with L-HS compared with both patients with H-HS and HC (Figure 1, C and D). Conversely, Trp levels were decreased in patients with L-HS, compared with both patients with H-HS and HC (Figure 1, C and D). Altogether, these data revealed constitutive alterations in HS skin metabolism, with accumulation of Kyn and Quin in lesions suggesting a local activation of Trp catabolism via the Kyn pathway.

### Distinctive and selective induction of Kyn pathway enzymes in HS skin.

**Figure 2:**
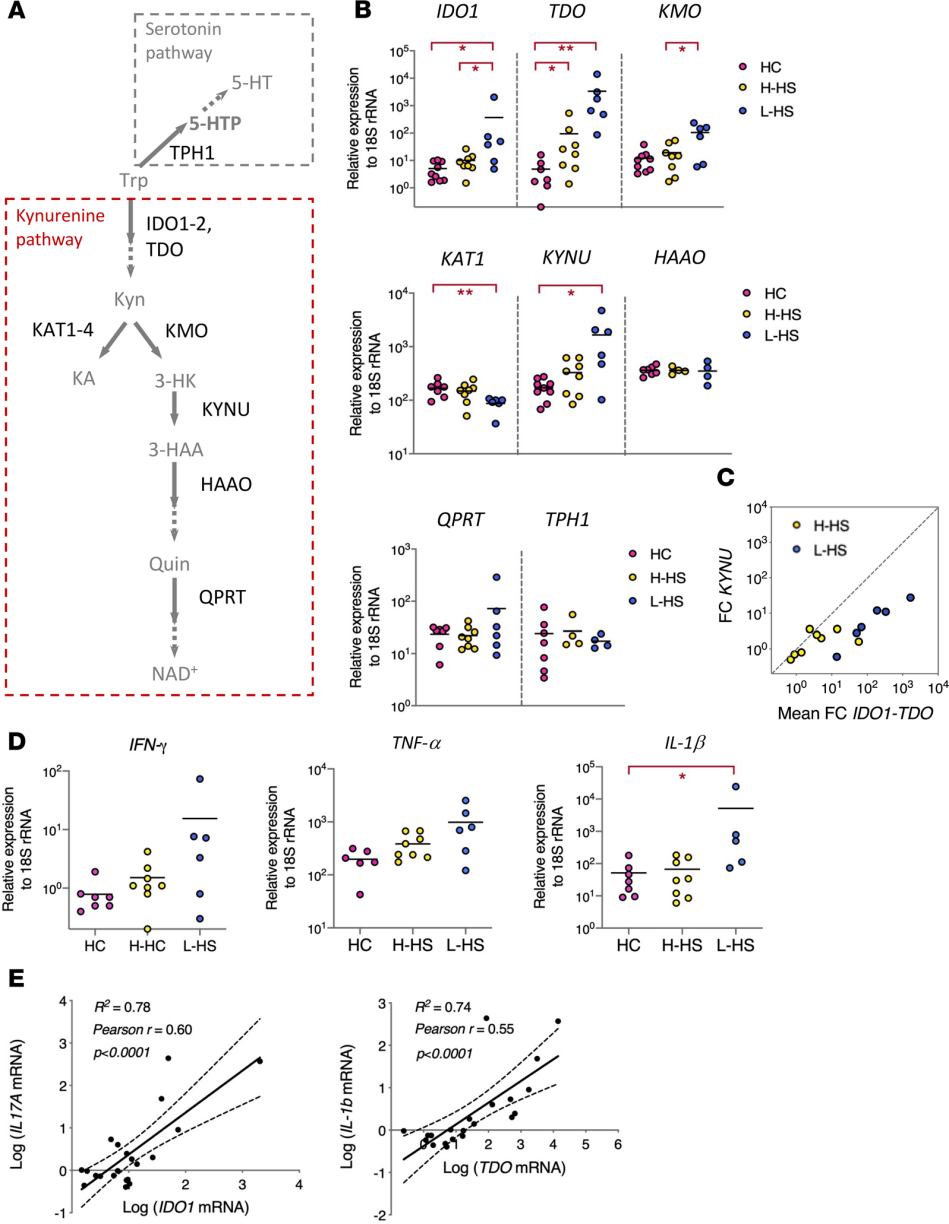
Distinctive and selective induction of kynurenine pathway enzymes in HS skin. (**A**) Enzymatic steps in the kynurenine and serotonin pathways of Trp catabolism. (**B**) Relative expression of kynurenine pathway enzymes in skin samples from HC and patients with HS. Data are shown as scatter dot plots with means. *P < 0.05, **P < 0.01 by Mann-Whitney *U* test for comparisons with HC and Wilcoxon matched-pairs test for comparisons of L-HS with matched H-HS. (**C**) Fold changes (FC) in expression of *IDO* and *TDO* (mean FC of the 2 genes) and *KYNU* are shown for H-HS (yellow) and L-HS (blue) skin samples. (**D**) Relative expression of cytokines in skin samples from HC and patients with HS, shown as scatter dot plots with means. *P < 0.05, **P < 0.01 by Mann-Whitney *U* test for comparisons with HC and Wilcoxon matched-pairs test for comparisons of L-HS with matched H-HS. (**E**) Correlation between *IDO1/IFN-γ* and *TDO/IL-1β* gene expression. Linear regression plots are shown, with R squared (R^2^), Pearson coefficient (r), and statistical significance. 5-HT, serotonin.

To determine whether the observed alterations in Trp metabolism were associated with changes in expression of Kyn pathway enzymes, a new cohort of patients with Hurley stage I HS and HC was recruited for transcriptomic analysis of skin biopsies (Supplemental Table 1). The first step of Trp degradation by the Kyn pathway is regulated by 3 rate-limiting enzymes: indoleamine 2,3-dioxygenase 1 (IDO1), IDO2, and Trp 2,3-dioxygenase (TDO) (Figure 2A). While *IDO2* was poorly expressed in all skin samples (data not shown), *IDO1* and *TDO* transcripts were both detected, and the 2 genes were more highly expressed in patients with L-HS than in HC (Figure 2B). Downstream of IDO1 and TDO, Kyn can be converted into 3-hydroxykynurenine (3-HK) by KMO or kynurenic acid (KA) by KAT enzymes (Figure 2A). Expression of *KMO* was augmented in patients with L-HS, while that of *KAT1* was decreased (Figure 2B), suggesting that Kyn is preferentially metabolized into Quin in HS skin lesions. Downstream of KMO, expression of *KYNU* (converting 3-HK into hydroxyanthranilic acid [3-HAA]) was also augmented in patients with L-HS compared with HC (Figure 2, A and B). In contrast, expression of *HAAO* (immediately downstream of KYNU) and *QPRT* (converting Quin into a NAD precursor) was not significantly modulated (Figure 2, A and B). The potent induction of *IDO1*, *TDO*, *KMO*, and *KYNU* without concomitant upregulation of *KAT1* and *QPRT* was thus consistent with the decreased levels of Trp and the accumulation of Kyn and Quin in HS skin lesions (Figure 1D).

Previous work has identified *KYNU* as one of the most upregulated genes in lesional skin of patients with psoriasis and atopic dermatitis (31–34), and preferential upregulation of *KYNU* over *IDO1* and *TDO* corelated with disease severity in these settings (35). In HS skin, *IDO1* and *TDO* were more highly expressed than *KYNU,* particularly in lesions (Figure 2C), indicating a distinct activation pattern of the Kyn pathway in this context that is not generically related to skin inflammation.

In addition to its catabolism to NAD, Trp can serve as a precursor for the neurotransmitter serotonin, via conversion into 5-HTP by Trp hydroxylase 1 enzyme (TPH1) (Figure 2A) (36). Our metabolomic analysis did not detect any product of the serotonin pathway, and *TPH1* was comparably expressed in skin from patients with HS and controls (Figure 2B), suggesting that this pathway was not modulated in HS skin. Together, our data in Figure 1 and Figure 2, A and B, thus revealed a distinctive and selective induction of the Kyn pathway of Trp catabolism in HS skin lesions.

IDO1 and TDO are important links between the Kyn pathway and inflammation, because their production is initiated by proinflammatory cytokines. IFN-γ is a powerful driver of *IDO1* gene expression, and IFN-γ–mediated induction of *IDO1* can be potentiated by other cytokines, such as TNF-α or IL-1β, while IL-1β itself is a *TDO* inducer (37, 38). Although statistical significance was not reached, expression of both *IFN-γ* and *TNF-α* tended to increase in lesions, and *IL-1β* expression was significantly augmented in patients with L-HS, compared with HC (Figure 2D). Notably, there was a strong positive correlation between *IDO1* and *IFN-γ* on the one hand and *TDO* and *IL-1β* on the other (Figure 2E), suggesting that IFN-γ and IL-1β produced by infiltrating immune cells locally activate Trp catabolism via transcriptional induction of *IDO1* and *TDO*.

### Immune infiltrates and dermal fibroblasts both contribute to Quin production in HS skin.

**Figure 3:**
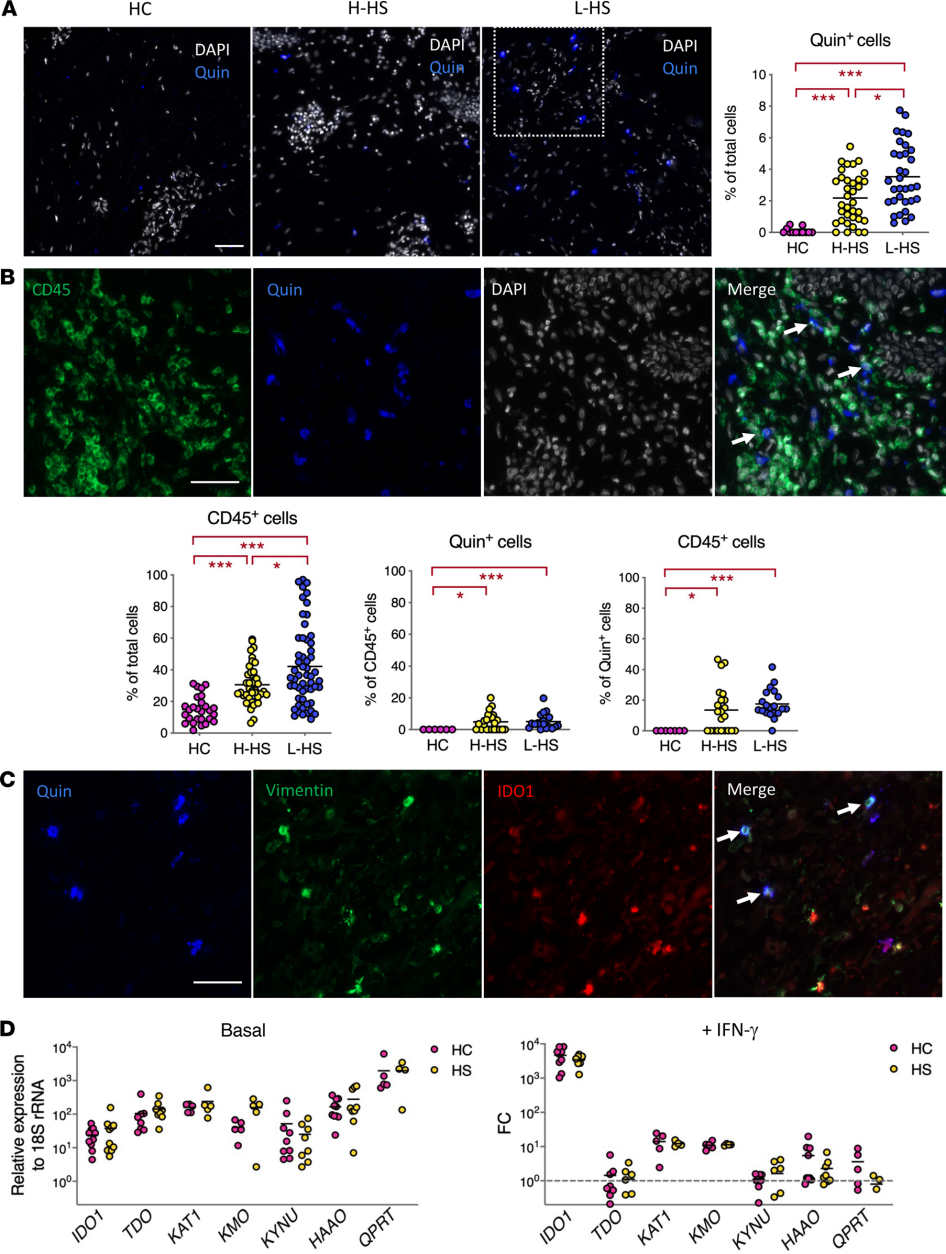
Immune infiltrates and dermal fibroblasts jointly contribute to Quin production in HS skin. (**A**) DAPI and Quin staining of representative HC, H-HS, and L-HS dermis sections, with mean incidence of Quin^+^ cells, relative to total DAPI^+^ cells, as measured on >5 skin sections from 3 HC and 4 patients with HS. (**B**) CD45, Quin, and DAPI staining of a representative L-HS dermis section, with arrows pointing to Quin^+^CD45^+^ immune cells. Quantification of CD45^+^ cells relative to total DAPI^+^ cells, CD45^+^ cells among Quin^+^ cells, and Quin^+^ cells among CD45^+^ cells. (**C**) Zoomed view of the area depicted in **A**, following Quin, vimentin, and IDO1 staining, with arrows pointing to Quin^+^vimentin^+^ IDO1^+^ cells. (**D**) Relative levels of kynurenine pathway enzyme transcripts in primary fibroblasts from 5 HC and 5 patients with HS in resting conditions (left). Fold change (FC) in gene expression following a 24-hour treatment with IFN-γ (2.5 ng/mL), relative to unstimulated controls (right). Scale bar: 50 μm. Scatter dot plots with means. *P < 0.05, **P < 0.01, ***P < 0.001 by Mann-Whitney *U* test, with Benjamini-Hochberg correction for multiple comparisons.

We next used multicolor immunohistochemistry to determine which cells were responsible for Quin production in HS skin. No Quin staining was detected in the epidermis of patients with HS or controls (data not shown). However, in line with our metabolomic data, Quin^+^ cells were found in the dermis of HS skin, and lesions contained a significantly higher number of Quin^+^ cells compared with nonlesional and normal skin. Notably, the frequency of Quin^+^ cells in H-HS skin was superior to that in HC (Figure 3A), further indicating that Quin production is altered in HS skin in the absence of lesion.

Since cytokine-induced expression of IDO can occur in immune cells (37), the production of Quin by these cells was investigated. Lesional skin, and nonlesional skin to a lesser extent, displayed significant infiltration of CD45^+^ hematopoietic, cells and a fraction of these cells stained positive for Quin (Figure 3B). However, CD45^+^ hematopoietic cells only represented <20% Quin^+^ cells in L-HS skin (Figure 3B), and >50% of Quin^+^ cells in L-HS skin were found to express the fibroblast marker vimentin (Figure 3C). Dermal fibroblasts are known to degrade Trp in response to stimulation with IFN-γ via induction of IDO1 (39). Consistent with this finding, Quin^+^vimentin^+^ cells in L-HS skin stained positive for IDO1 (Figure 3C). To determine if fibroblasts of patients with HS were intrinsically prone to degrading Trp, we generated primary fibroblasts from patients and controls (Supplemental Table 1) and analyzed their expression of Kyn pathway enzymes. There was no significant difference in basal expression of *IDO1*, *TDO*, *KAT1*, *KMO*, *KYNU*, *HAAO*, and *QPRT* in fibroblasts of patients with HS or controls, and HS fibroblasts responded normally to IFN-γ, TNF-α, and IL-1β stimulation (Figure 3D and Supplemental Figure 1). We concluded that enhanced production of Quin in patients with L-HS does not result from a constitutive activation of the Kyn pathway, but rather from cytokine-driven induction of Trp catabolism in dermal fibroblasts and a subset of infiltrating immune cells.

### Plasma Trp levels are decreased in patients with HS.

**Figure 4:**
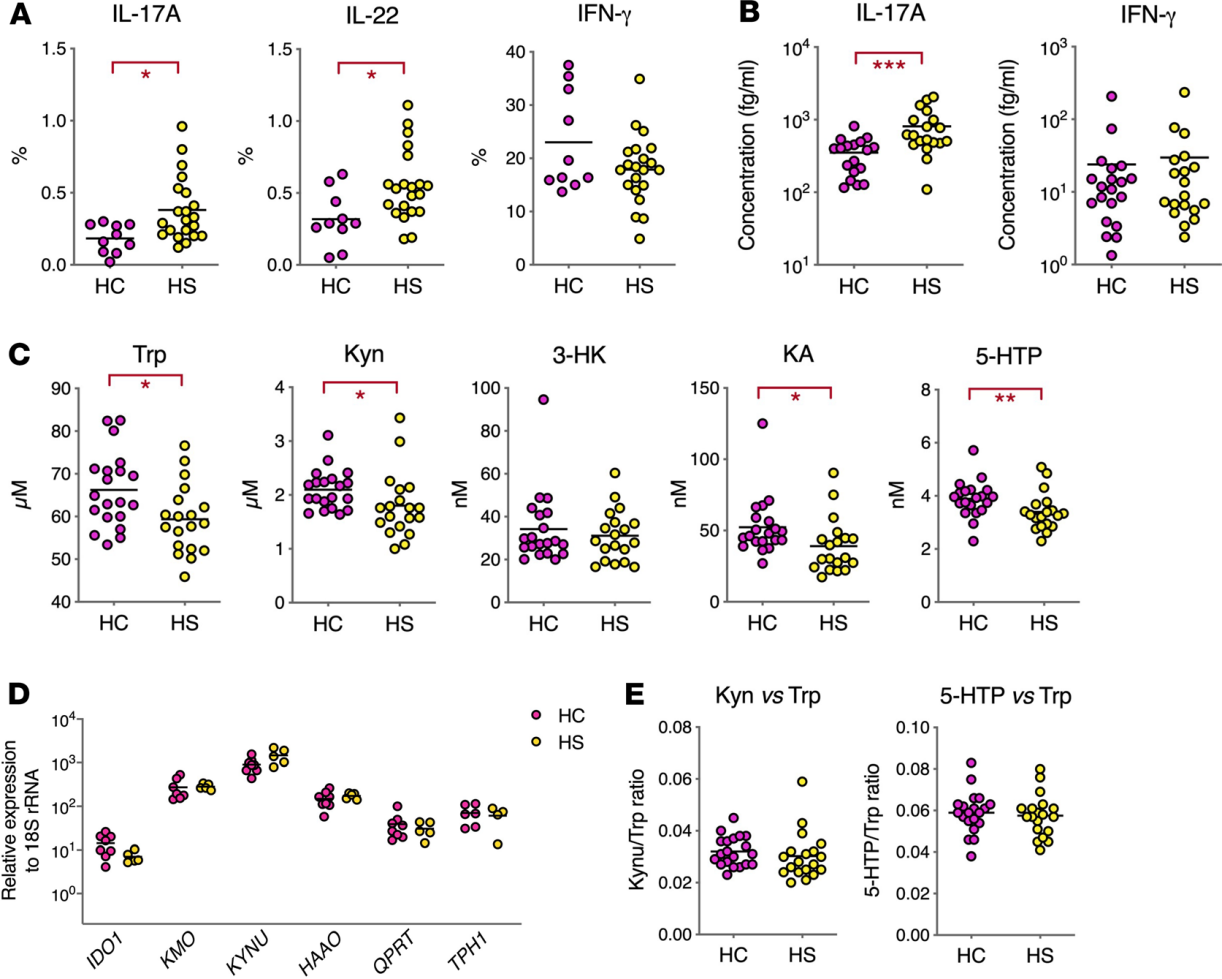
Plasma Trp levels are decreased in patients with HS. (**A**) Frequency of cytokine-producing blood cells, relative to total T cells, following a 3-hour stimulation with PMA/ionomycin. (**B**) Cytokine concentrations in plasma from HC and patients with HS. (**C**) Comparison of Trp metabolite concentrations in plasma of HC and patients with HS. (**D**) Relative expression of kynurenine pathway enzymes in PBMCs from HC and patients with HS. (**E**) Ratios of Kyn or 5-HTP to Trp in plasma. Data are shown as scatter dot plots with means. *P < 0.05, **P < 0.01, ***P < 0.001 by Mann-Whitney *U* test.

The data in Figures 1, 2, and 3 revealed alterations in the Trp metabolism of HS skin that are associated with the local production of inflammatory cytokines. To determine if these immune and metabolic perturbations extended beyond skin lesions, we examined the production of Kyn pathway–inducing cytokines by peripheral blood lymphocytes. Consistent with previous studies, peripheral blood lymphocytes from patients with HS showed an increased frequency of IL-17A– and IL-22–producing T cells, while IFN-γ–producing T cells were unaltered (Figure 4A and Supplemental Figure 2A). While IL-17A protein levels were significantly augmented in the plasma of patients with HS, IFN-γ levels remained unchanged (Figure 4B). Moreover, TNF-α and IL-1β plasma levels were below the detection limit in all samples (data not shown), thus excluding a systemic inflammatory syndrome in the patients studied.

We next investigated if Trp metabolism was altered at the systemic level by quantifying Trp metabolites in the plasma. Plasma levels of Trp, Kyn, KA, and 5-HTP were significantly lower in patients with HS, compared with controls (Figure 4C). The decrease in plasma levels of Trp metabolites was not due to differential induction of the Kyn or serotonin pathways in PBMCs, as the basal expression of *IDO1*, *KMO*, *KYNU*, *HAAO*, *QPRT*, and *TPH1* was comparable in PBMCs from patients with HS and controls (Figure 4D). Moreover, the Kyn/Trp and 5-HTP/Trp ratios were unchanged (Figure 4E). In conclusion, this analysis revealed abnormally low levels of circulating Trp in patients with HS that were not accompanied by a systemic inflammatory state.

### Activation of AHR is defective in HS skin lesions.

**Figure 5:**
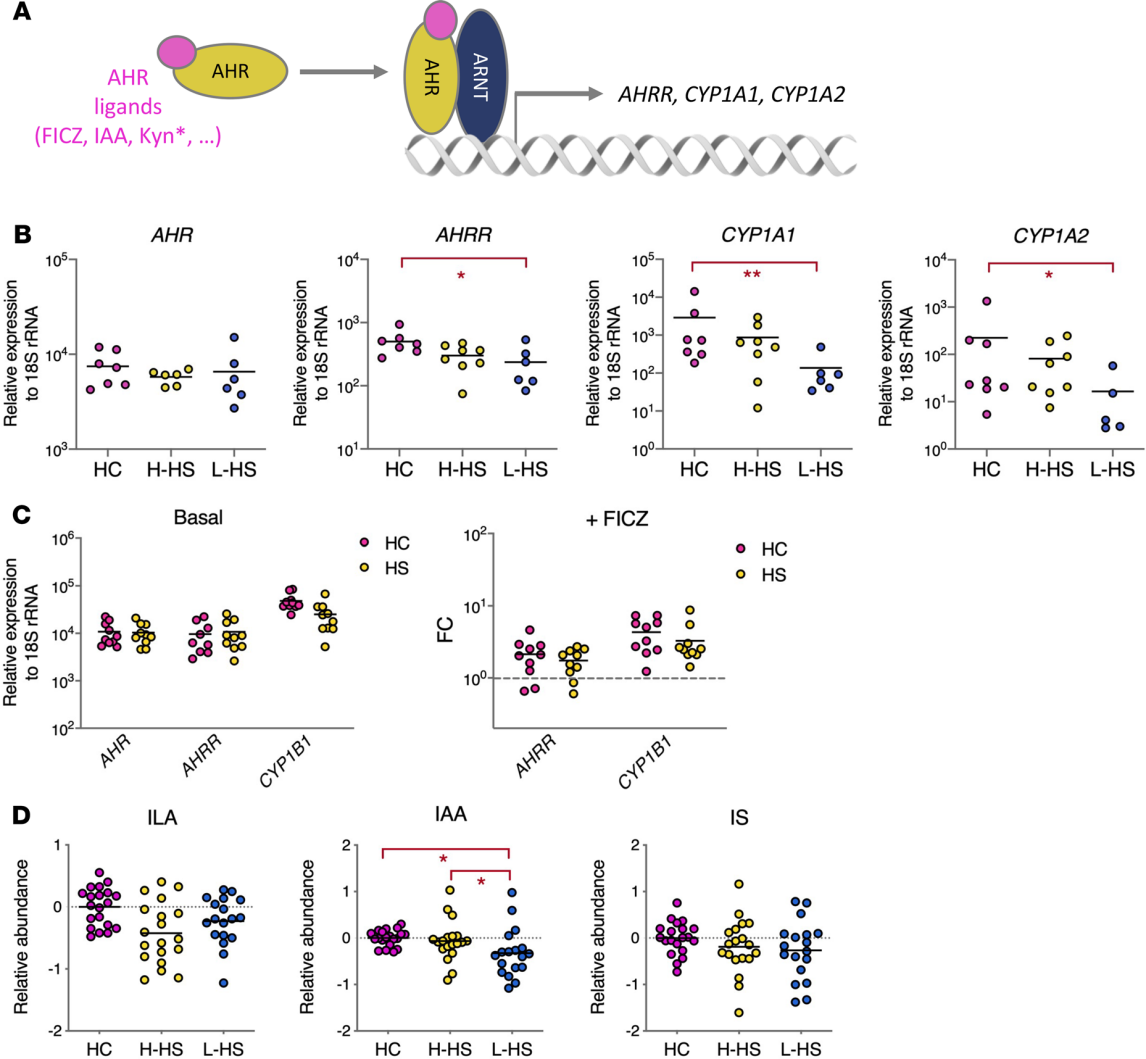
Activation of AHR is defective in HS skin lesions. (**A**) The AHR pathway. Upon ligand binding, AHR translocates into the nucleus where association with ARNT and interaction with specific genomic sequences induce the transcription of target genes (asterisk indicates active in supraphysiological concentrations). (**B**) Relative levels of AHR and AHR-controlled transcripts in skin samples from HC and patients with HS. *P < 0.05, **P < 0.01 by Mann-Whitney *U* test for comparisons with HC, and Wilcoxon matched-pairs test for comparisons of L-HS with matched H-HS. (**C**) Relative expression of AHR-controlled transcripts in fibroblasts from HC and patients with HS in resting (left) or FICZ-stimulated (right) conditions. Data are from 2 independent experiments, with fibroblasts from 5 HC and 5 patients with HS. (**D**) Relative levels of indole metabolites in skin samples from HC and patients with HS. *P < 0.05, **P < 0.01, by Welch’s 2-sample *t* test for comparisons with HC and matched pairs *t* test for comparisons of L-HS with matched H-HS, with Benjamini-Hochberg correction for multiple comparisons.

Our observation that Trp levels were decreased in HS skin lesions (Figure 1D) suggested that Trp catabolism by resident bacteria may be altered. This essential amino acid is indeed an energy source for the microbiota, which modulates local immune responses through production of indole and derivatives (40). Microbiota-derived indole metabolites include ligands of the aryl hydrocarbon receptor (AHR), a transcription factor translating metabolic signals into cell type–specific gene expression programs at mucosal surfaces (41) (Figure 5A). All cells in the skin express AHR, and physiological activation of AHR in keratinocytes and dermal fibroblasts is critical for the regulation of skin inflammatory responses (40). To determine whether AHR signaling was altered in HS skin, we compared the expression of *AHR* and *AHR*-dependent genes in healthy and diseased skin samples. While *AHR* expression was comparable in HC and patients with H-HS and L-HS, that of AHR target genes *AHRR*, *CYP1A1*, and *CYP1A2* was significantly decreased in patients with L-HS, compared with HC (Figure 5B). To see if defective activation of AHR in HS skin lesions was due to intrinsic defects in AHR signaling, we next examined the integrity of this pathway in fibroblasts of patients with HS. Figure 5C shows that patients with HS- and HC-derived fibroblasts displayed comparable expression of *AHR*, *AHRR*, and *CYP1B1* in the resting state and, when stimulated with the prototypical AHR agonist FICZ, displayed comparable induction of *AHRR* and *CYP1B1*. This suggested that decreased activation of AHR in lesional skin is not due to intrinsic defects, but rather caused by locally impaired production of AHR agonists. Known agonists of AHR include Kyn, a low-affinity ligand whose physiological relevance is doubtful (42), and indole derivatives originating from bacterial degradation of Trp. Among bacterial metabolites of Trp, our metabolomic analysis detected indole-3-acetic acid (IAA), indole-3-lactic acid (ILA), and indoxyl-sulfate (IS), of which IAA is a recognized AHR agonist (43). Notably, IAA levels were significantly decreased in patients with L-HS, compared with patients with H-HS and HC (Figure 5D). Therefore, the defective activation of AHR in HS skin lesions coincides with an impaired production of AHR agonist IAA by the skin microbiota.

## Discussion

In this report, we reveal that patients with Hurley stage I HS display local and systemic alterations in Trp metabolism. A selective and potent induction of the Kyn pathway of Trp degradation was detected in HS skin lesions. Based on our histologic (Figure 3, C and D) and transcriptomic (Figure 2, B and D, and Supplemental Figure 1) data, we propose that induction of this pathway results from the recruitment and activation of immune cells that release IFN-γ and IL-1β, which jointly contribute to triggering expression of *IDO1* and *TDO* in dermal fibroblasts and a subset of infiltrating immune cells. The alterations in Trp metabolism that we observed in HS skin differ from those previously reported in other inflammatory skin diseases, in magnitude and profile. In HS skin lesions, transcriptional activation of the Kyn pathway was dominated by expression of *IDO1* and *TDO*, leading to local accumulation of Kyn and Quin (Figures 1 and 2). These 2 enzymes were either not modulated or only mildly upregulated in published transcriptomes of atopic and psoriatic skin (31–34), and the skin levels of Kyn pathway metabolites were unchanged in these settings (44, 45). While *KYNU* is one of the top upregulated genes in both atopic dermatitis and psoriasis, induction of *KYNU* was lower than that of *IDO1/TDO* in HS skin (Figure 2C). In contrast to that of *IDO1*, *KYNU* expression is optimally triggered by the combination of IFN-γ and TNF-α (35). Its relatively lower induction in HS skin lesions may reflect a comparatively less important production of *TNF-α* in studied patients. Since TNF-α potentiates both the IFN-γ–driven induction of *IDO1* and expression of *KYNU*, its upregulation in more severe HS lesions may further activate the Kyn pathway. It is important to note that our study is the first to our knowledge to focus on a clinically homogenous group of patients with Hurley stage I. This might explain differences with previous studies, which involved patients with variable and often severe HS lesions (10, 12, 14, 15). Our immune profiling of PBMCs from patients with HS highlighted higher frequencies of NK cells expressing granzyme B and perforin and producing IFN-γ upon stimulation (Supplemental Figure 2, B–D). Since NK cells are potent producers of IFN-γ that were identified in HS lesions (Supplemental Figure 2E), these data suggest that NK cells are primed for effector functions in patients with HS and that their local activation may contribute to *IDO1* induction in lesional skin.

Importantly, our analysis of HS plasmas also revealed for the first time to our knowledge abnormally low levels of circulating Trp (Figure 4C). Whether depletion in plasma Trp results from increased degradation in HS skin or from metabolic dysregulations in other organs is an open question. Since Trp is an essential amino acid, our data suggest that intestinal absorption or liver catabolism of Trp may be altered in patients with HS.

We found that CD45^+^Quin^+^ cell numbers, Quin levels, and *TDO* transcripts were elevated in clinically normal skin of patients with HS (Figure 1D, Figure 2B, and Figure 3B). This suggests that inflammation and the Kyn pathway are induced throughout HS skin, with the caveat that H-HS skin samples were harvested in the periphery of lesions in the present work. Recent metagenomic studies have revealed a distinctive dysbiosis in clinically normal skin of patients with HS, marked by the expansion of *Corynebacterium* spp and anaerobes (46). In line with these findings, our analysis of the skin microbiota using 16S ribosomal RNA sequencing showed that the microbiome of H-HS skin differed significantly from that of HC by the increased abundance of *Corynebacterium* spp and anaerobic bacteria (Supplemental Figure 3, A and B). Expansion of these bacterial species may contribute to stimulation of inflammation and Trp degradation at a preclinical stage of disease.

Overactivation of the Kyn pathway could contribute to the underlying pathogenic mechanisms of HS in various ways. First, decreased availability of Trp in the skin may provide Trp-independent pathobionts such as *Staphylococcus aureus* with a selective growth advantage. A recent analysis of skin microbial communities coupled to cutaneous gene expression in patients with atopic dermatitis or psoriasis found that dense colonization by *S*. *aureus* correlates with increased expression of *TDO*, *KYNU*, and *KMO* (31). While our analysis of HS skin microbiota failed to identify *Staphylococci* to species level, *S*. *aureus* was previously identified as an important colonizer of HS skin lesions, with a prevalence rate reaching up to 56% (47). Our findings suggest that activation of the Kyn pathway in HS skin lesions may favor the expansion of this pathobiont.

Second, decreased Trp availability may lower the production of AHR ligands by the skin microbiota, thereby altering AHR-mediated regulation of inflammation. Among bacterial AHR agonists, our metabolomic analysis detected the *Lactobacillus* product IAA. Our observation that IAA was decreased in lesional skin, while Kyn and Quin were increased instead, indicates that Trp degradation by *Lactobacilli* was downmodulated. In support of this hypothesis, the relative abundance of *Lactobacillus* spp was significantly lower in L-HS compared with HC (Supplemental Figure 3B). Recent studies of atopic skin have reported a selective decrease in skin levels of the IAA product indole-3-aldehyde (IAId) (45). Notably, treating with IAId topically or orally alleviated skin inflammation in a mouse model of atopic dermatitis in a AHR-dependent manner (45). Altogether, these data strongly support the view that defective production of AHR agonists by resident bacteria contributes to immune dysregulation in HS skin lesions.

Finally, depression and anxiety are common conditions in patients with HS (2). Overactivation of the IDO1 pathway in patients with chronic inflammatory diseases, or in patients treated with type I IFN, was proposed to cause depression via central serotonin depletion or increased production of neurotoxic Quin in the brain (36). Plasma levels of Trp, Kyn, and 5-HTP were significantly reduced in patients with HS (Figure 4C). Since these Trp metabolites are able to cross the blood-brain barrier, dysregulated Trp metabolism in HS skin could impact brain chemistry and predispose to depressive symptoms.

In conclusion, this study reports for the first time to our knowledge local and systemic alterations in the Trp metabolism of patients with HS. Dysregulation of Trp catabolism at the host-skin microbiota interface provides a potential mechanism for the diverse clinical manifestations of HS. In the absence of animal models for HS, further investigations in patients will be needed to elucidate the molecular origin of these metabolic defects. Trp degradation via the Kyn pathway has immunosuppressive effects, operating through the formation of catabolites inducing a regulatory phenotype in T cells and DCs (41). However, there is accumulating evidence that in barrier organs, such as the gut and the skin, exacerbated IDO activity may downregulate AHR agonist production by the microbiota, thereby promoting chronic inflammation (36, 40). While Trp metabolites generated by the gut microbiota are now well characterized (48), little is known about Trp metabolism by skin-resident bacteria and their capacity to generate immunomodulatory AHR ligands. Correcting AHR signaling via administration of AHR agonist FICZ or transplantation of Trp-metabolizing *Lactobacillus* strains proved to be effective at reducing intestinal inflammation in a mouse model of inflammatory bowel disease (49), and FICZ also attenuated skin inflammation in a mouse model of psoriasis (50). Studies on the effects of topically applied bacterial products and skin bacterial transplants have yielded promising results in animal models and human studies of atopic dermatitis (45, 51). Our study suggests that such approaches may help restore immune homeostasis in HS skin.

## Methods

### Study design and harvested specimens.

The characteristics of patients with HS and healthy individuals recruited for this study are listed in Supplemental Table 1. Specific inclusion criteria included age ≥18 years and, for patients, diagnosis of active Hurley stage 1 HS. Noninclusion criteria for both patients and healthy subjects were as follows: pregnancy, antibiotic or immunosuppressive treatment during the past month, chronic inflammatory disease, cancer, hematological malignancy, and contraindication to biopsy. For healthy subjects, a progressive skin disease, or any personal or familial history of chronic inflammatory disease, constituted additional noninclusion criteria.

### Harvested specimens.

In patients with HS, punch biopsies (4 mm diameter) were harvested in lesional skin and clinically normal, perilesional skin (approximately 5 cm from the inflamed nodule). Biopsy specimens from HC were harvested in the armpit area. Immediately after harvesting, biopsies were snap frozen at –80°C until metabolomic, transcriptomic (in RNAlater), and histologic analysis. Primary fibroblasts were derived from healthy perilesional skin (patients with HS) or normal skin from surgical margins (controls) as described previously (52). Primary fibroblasts were cultivated in DMEM (Gibco) supplemented with 10% fetal calf serum and 1% Penicillin-Streptomycin (MilliporeSigma) and stimulated for 24 hours with 100 ng/mL of the AHR agonist FICZ or with IFN-γ (2.5 ng/mL) in the presence or absence of TNF-α (100 ng/mL). Venous blood (2 × 10 mL) was collected, and, within 1 hour, PBMCs and plasmas were separated by Ficoll and frozen at –196°C/–80°C, respectively, until proteomic flow cytometric studies.

### Metabolomic profiling of skin biopsies.

Skin samples were prepared using the automated MicroLab STAR system (Hamilton Company) for global untargeted metabolic profiling by Metabolon Inc. Proteins were precipitated with methanol under vigorous shaking for 2 minutes (Glen Mills GenoGrinder 2000) followed by centrifugation. The resulting extract was divided into 5 fractions: 2 for analysis by 2 separate reverse phase/ultraperformance liquid chromatography–mass spectrometer/mass spectrometer (RP/UPLC-MS/MS) methods with positive ion mode electrospray ionization (ESI), 1 for analysis by RP/UPLC-MS/MS with negative ion mode ESI, 1 for analysis by hydrophilic interaction chromatography/UPLC-MS/MS (HILIC/UPLC-MS/MS) with negative ion mode ESI, and 1 sample reserved for backup. Samples were placed briefly on a TurboVap (Zymark) to remove the organic solvent. UPLC-MS/MS analysis of the resulting samples used a Waters ACQUITY UPLC and a Thermo Scientific Q-Exactive high resolution/accurate MS interfaced with a heated ESI (HESI-II) source and Orbitrap mass analyzer operated at 35,000 mass resolution. The sample extract was dried and then reconstituted in solvents compatible with each of the 4 methods. Each reconstitution solvent contained a series of standards at fixed concentrations to ensure injection and chromatographic consistency. One aliquot was analyzed using acidic positive ion conditions, chromatographically optimized for more hydrophilic compounds. In this method, the extract was gradient eluted from a C18 column (Waters UPLC BEH C18-2.1 × 100 mm, 1.7 μm) using water and methanol, containing 0.05% perfluoropentanoic acid (PFPA) and 0.1% formic acid (FA). Another aliquot was also analyzed using acidic positive ion conditions, however it was chromatographically optimized for more hydrophobic compounds. In this method, the extract was gradient eluted from the same aforementioned C18 column using methanol, acetonitrile, water, 0.05% PFPA, and 0.01% FA and was operated at an overall higher organic content. Another aliquot was analyzed using basic negative ion optimized conditions using a separate dedicated C18 column. The basic extracts were gradient eluted from the column using methanol and water with 6.5 mM ammonium bicarbonate, pH 8. The fourth aliquot was analyzed via negative ionization following elution from a HILIC column (Waters UPLC BEH Amide 2.1 × 150 mm, 1.7 μm) using a gradient consisting of water and acetonitrile with 10 mM ammonium formate, pH 10.8. The MS analysis alternated between MS and data-dependent MSn scans using dynamic exclusion. The scan range varied slightly between methods but covered 70–1000 *m/z*. Subsequent bioinformatic analysis used Metabolon proprietary tools for raw data extraction, peak identification software, QC, and compound identification. A total of 500 biochemicals were identified in our data set. Metabolite data were log_2_ transformed and normalized using Pareto scaling. The statistical analysis was performed on R software using the limma package (53). FDRs were calculated using the Benjamini-Hochberg method (Supplemental Table 2).

### Quantitative assay of Trp metabolites in serum.

Blood levels of 5 metabolites (Trp, Kyn, 3-HK, KA, and 5-HTP) were assessed in serum samples by high-performance liquid chromatography coupled to tandem mass spectrometry, using methodologies adapted from ref. 54.

### PBMC studies.

Ficoll-isolated PBMCs were activated with PMA (10 ng/mL; MilliporeSigma) plus ionomycin (1 μg/mL; MilliporeSigma) over 3 hours at 37°C in the presence of BD GolgiPlug (brefeldin A; BD Biosciences) and BD GolgiStop (monensin; BD Biosciences). Flow cytometry analysis of T cells and NK cells was performed using the following antibodies: biotinylated anti-human CD1a (HI149; BioLegend), CD3 (OKT3; Invitrogen), CD14 (61D3; Miltenyi Biotec), CD19 (HIB19; BioLegend), CD34 (4H11; BioLegend), CD123 (6H6; BioLegend), CD203c (FR3-16A11; BioLegend), CD303 (AC144; BioLegend), TCRαβ (IP26; Invitrogen), TCRγδ (B1; Invitrogen) and FcεRIα (AER-37; BioLegend) Abs in combination with the streptavidin BV711 (BioLegend); and conjugated anti-human CD5 Alexa Fluor 700 (L17F12; BioLegend), CD7 BV650 (M-T701; BD Biosciences), CD56 BUV737 (NCAM16.2; BD Biosciences), CD127 PE-Cy7 (eBioRDR5; Invitrogen), NKp44 BB515 (p44-8; BD Biosciences), CD69 PE (FN50; Invitrogen), PD-1 BV786 (EH12.2H7; BioLegend), CD25 BUV563 (2A3; BD Biosciences), HLA-DR BUV661 (G46-6; BD Biosciences), CD45 BUV805 (HI30; BD Biosciences), EOMES PE-eFluor 610 (WD1928 Invitrogen), Perforin BV421 (dG9; BD Biosciences), Granzyme B PE-CF594 (GB11; BD Biosciences), IFN-γ BUV395 (B27; BD Biosciences), IL-17A BV570 (BL168; BD Biosciences) and IL-22 PE (22URTI; Invitrogen). Fc receptors were blocked using IgG from human serum (MilliporeSigma). Surface membrane staining was performed in Brilliant Stain Buffer (BD Biosciences). Transcription factors and cytokines were stained using the Foxp3 staining buffer set (Thermo Fisher Scientific) according to the manufacturer’s instructions. The fixable viability dye eFluor 506 (Thermo Fisher Scientific) was used to exclude dead cells. Samples were acquired on a Symphony A5 (BD Biosciences) using FACSDiva 8 and analyzed with FlowJo 10 (BD Biosciences).

### Quantitative reverse-transcription PCR.

Total RNA was extracted from pulverized skin biopsies or cell pellets with Qiazol lysis reagent (QIAGEN) and then purified using the QIAGEN RNeasy Mini Kit and digested with RNase-Free DNase set (QIAGEN, 79254) for 15 minutes at room temperature. First-strand cDNA was synthesized from 400 ng total RNA with the high-capacity cDNA reverse transcription kit (Applied Biosystems, 4368814). Expression was quantified using Power SYBR Green PCR Master Mix (Applied Biosystems, 4367659) and gene-specific primers (Supplemental Table 3). Amplification was performed from a 5 ng cDNA template in a final volume of 20 μL in a 96-well PCR plate. Amplification conditions were as follows: 2 minutes at 50°C, 10 minutes at 95°C, followed by 40 cycles of 15 seconds at 95°C and 1 minute at 60°C on a Quant Studio 3 Real-time PCR System (Applied Biosystems). Results were normalized by relative expression using 18S rRNA as an endogenous control.

### Histology.

Frozen skin biopsies were fixed overnight at 4°C with 4% paraformaldehyde in phosphate buffer. Tissues were cryopreserved by immersion in 30% sucrose for 24 hours and then embedded in optimal cutting temperature compound (Tissue-Tek O.C.T, Sakura) and stored at –80°C until sectioning. Immunostaining was performed on 8 μM sections obtained with a Cryostat (CM3050 S, Leica) on SuperFrost Plus adhesion slides (Thermo Fisher Scientific). Nonspecific staining was blocked for 1 hour with 0.1% Triton X-100 and 10% fetal calf serum before overnight incubation with primary antibodies at 4°C. Primary antibodies included rabbit polyclonal anti-Quinolinic acid (Abcam, ab37106), mouse monoclonal anti-human CD45 (2D1) (BD Biosciences, 347460), rabbit polyclonal anti-human CD3ε (Dako, A0452), sheep polyclonal anti-human CD7 (R&D Systems, AF7579), mouse anti-IDO (1F8.2) (Merck, MAB10009), and polyclonal goat anti-vimentin (R&D Systems, AF2105). Donkey anti-rabbit A647 (Thermo Fisher Scientific, A-31573), anti-sheep A555 (Abcam, ab150177), anti-mouse A488 (Thermo Fisher Scientific, A-21202), anti-goat A488 (Thermo Fisher Scientific, A-11055), and anti-mouse A555 (Thermo Fisher Scientific, A-31570) were used as secondary antibodies. Images were acquired on an Axio Imager Z.2 (Zeiss) using the Imager Z.2 software and analyzed with the Icy open source platform (55).

### Plasma cytokine quantification.

IFN-γ and IL-17A concentrations in plasma were quantified by a high-sensitivity Simoa multiplex assay developed with Quanterix Homebrew kits, as previously described (56).

### 16S rRNA sequencing and analysis.

The V3-V4 region of bacterial 16S rDNA was PCR amplified with V3-340F (CCTACGGRAGGCAGCAG) and V4-805R (GGACTACHVGGGTWTCTAAT) barcoded primers. PCR products were cleaned using Ampure magnetic purification beads (Agencourt AMPure XP Kit), quantified with the QuantiFluor ONE dsDNA kit (Promega), and pooled in equal amounts of each PCR product. Library pools were loaded at 12 pM with a 15% PhiX spike for diversity and sequencing control onto a v3 300 bp paired end reads cartridge for sequencing on the Illumina MiSeq NGS platform. After removing reads containing incorrect primer or barcode sequences and sequences with more than one ambiguous base, a total of 2,905,540 reads (121,064 mapped reads on average) was recovered from the 24 studied samples. Bioinformatics analysis was performed as described in ref. 57. All read sequences were deposited in the Sequence Read Archive (SRA) (submission: SUB8079771/BioProject ID: PRJNA661179).

### Statistics.

GraphPad Prism software (version 6.0) was used for statistical comparisons and graphical representations. The statistical tests used are detailed in figure legends, including Welch’s 2-sample, 2-tailed *t* test, matched pairs 2-tailed *t* test, Benjamini-Hochberg correction for multiple comparisons, Mann-Whitney *U* test, Wilcoxon matched-pairs test, and Pearson coefficient. Comparisons of metabolite levels were performed by linear model regression using the limma package in R software (53) and comparisons between lesions and matched healthy skin controls (L-HS vs. H-HS) used a paired model, while comparisons between patients and HC were corrected for sex and age difference by including those factors as confounders in the model. A FDR was calculated using the Benjamini-Hochberg method to account for multiple comparisons in analyses of metabolomic and imaging data. Differences corresponding to FDR < 0.05 were considered significant.

### Study approval.

This biomedical research study was approved by the French Ethics Committee (Comité de Protection des Personnes, Paris, France), the competent health authority (Agence Nationale de Sécurité des Médicaments et des produits de santé, Saint-Denis, France), and the French Data Protection Agency (Commission Nationale de l’Informatique et des Libertés, Paris, France). Study participants were identified by number. They provided written informed consent to the research, including data and biospecimen collection, such as cutaneous biopsy sampling, before inclusion in the study.

## Author contributions

CD, JPDS, AN, and MD conceived and designed the study. CD, LGM, JDM, and JMD analyzed the data, and CD wrote the manuscript. SD and AH provided primary fibroblasts and skin samples from patients with HS and controls. MD, AN, and MNU conducted the recruitment of patients and healthy volunteers and the collection of human samples. JPDS and JMD designed and conducted the FACS analyses of PBMCs, with assistance from LGM. LGM, JDM, and VM designed and conducted all experiments using fibroblasts. LGM designed and conducted skin immunostaining studies, with assistance from AS. JDM analyzed metabolomics data. LB conducted the HPLC analysis of Trp metabolites in plasmas. DD supervised the Simoa analysis of plasma cytokines performed by VB, and PG designed and conducted 16S rRNA sequencing experiments and analysis.

## Supplementary Material

supplemental data

supplemental Table 2

## Version 1 09/24/2020

In-Press Preview

## Version 2 10/15/2020

Electronic publication
