# Supplementary material for: Dysregulation of tryptophan catabolism at the host-skin microbiota interface in hidradenitis suppurativa
Source: JCI Insight. 2020 Oct 15;5(20):e140598. doi: 10.1172/jci.insight.140598 (PMC7605522; doi:10.1172/jci.insight.140598)
Supplement: supplemental data [file jciinsight-5-140598-s104.pdf]

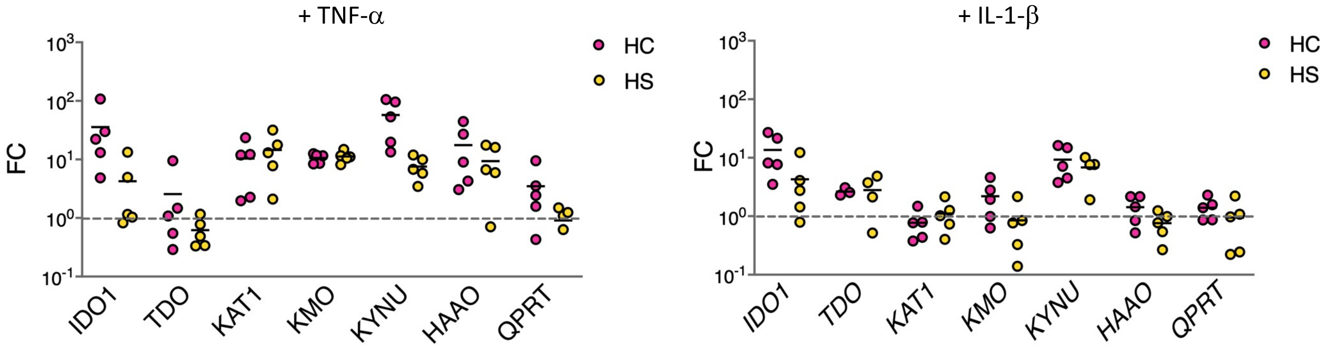

Figure S1. HS fibroblasts respond normally to *in vitro* stimulation with inflammatory cytokines. Fold change (FC) in expression of kynurenine pathway enzyme genes by primary fibroblasts from 5 HC and 5 HS patients following a 24h treatment with TNF-α (100 ng/ml) or IL-1β (2.5 ng/ml), relative to unstimulated controls. Data are shown as scatter dot plots with means.

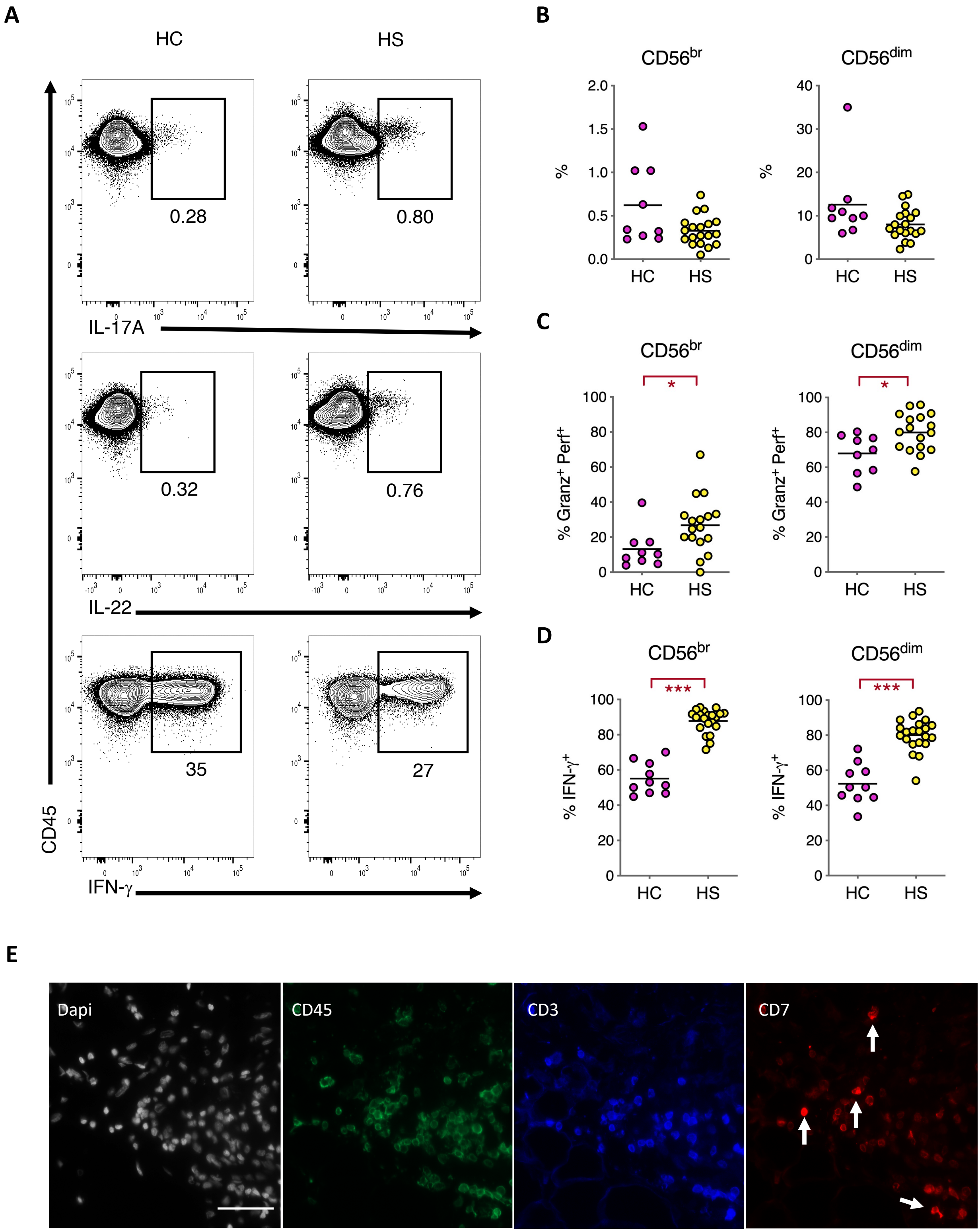

Figure S2. Immunological parameters differing between HS patients and HC controls. (A) Representative contour plots with percentages of IL-17A, IL-22 and IFN-γ producing T cells (gated as $CD3^+$ $CD5^+$ $CD45^+$) in HS patients and HC controls, following a 3h stimulation with PMA/Ionomycin. 100,000 event per contour plots are depicted for comparison. (**B**) Frequency of CD56br and CD56dim NK cells, amongst total lymphocytes, in HC and HS patients. (**C**) Proportion of CD56br and CD56dim NK cells co-expressing Granzyme B and Perforin. (**D**) Proportion of CD56br and CD56dim NK cells producing IFN-γ following a 3h stimulation with PMA/Ionomycin. Data are shown as scatter dot plots with means. *P<0.05, ***P<0.001 by Mann-Whitney U-test. (**E**) DAPI, CD45, CD3 and CD7 staining of a representative L-HS skin section, with arrows pointing to $CD3^-$ $CD7^+$ NK cells. Scale bar 50 µm.

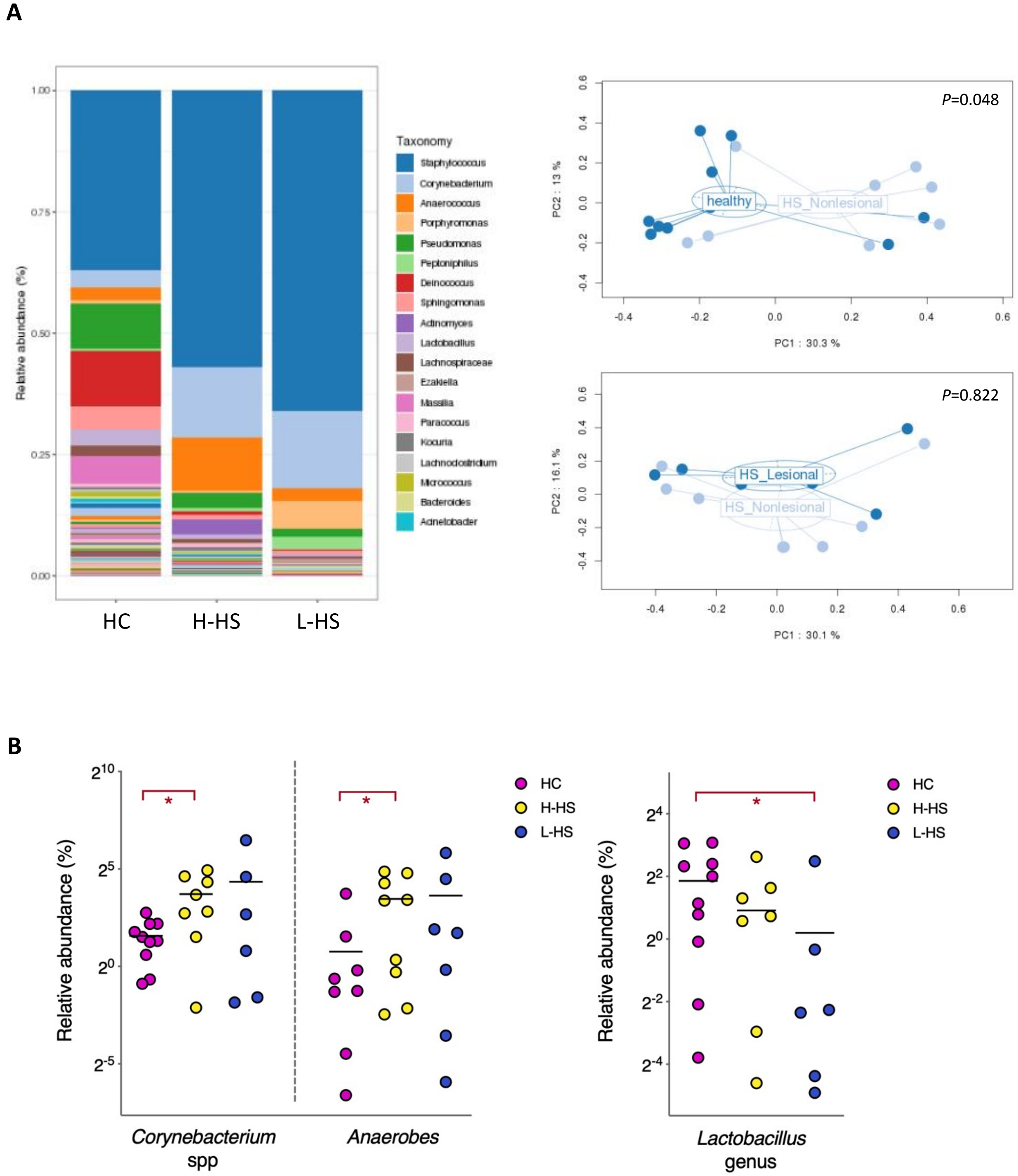

Figure S3. Changes in the bacterial composition of healthy and lesional skin microbiomes. (**A**) Genus distribution and PCA analysis of the bacterial species found in HC, H-HS and L-HS skin biopsies. *P* values by Permanova test. (**B**) Relative abundance of *Corynebacterium* spp, pooled anaerobes (*Prevotella*, *Porphyromonas*, *Actinomyces* and *Anaerococcus* spp) and *Lactobacillus* spp in HC, H-HS and L-HS skin samples. **P*<0.05, by Mann-Whitney U-test.

Table S1. Characteristics of study participants. (**A**) Metabolomic analysis of skin biopsies, assays of Trp metabolites and cytokines in plasma. (**B**) Transcriptomic analysis of skin biopsies and microbiota; *In 2 of the 8 HS patients, only H-HS skin samples were taken. (**C**) Primary fibroblasts.

A

	**Healthy controls**	**Patients with HS**
**Total**	20	19
**Female, No (%)**	12 (60)	16 (84)
**Age, mean (SD)**	46 (12)	32 (6)
**Puncture site**	Axilla	Axilla (5), Groin (8), Buttock (6)

B

	**Healthy controls**	**Patients with HS**
**Total**	9	8*
**Female, No (%)**	8 (89)	8 (100)
**Age, mean (SD)**	34 (7)	30 (8)
**Puncture site**	Abdomen (5), Leg (4)	Groin (4), Buttock (4)

C

	**Healthy controls**	**Patients with HS**
**Total**	5	5
**Female, No (%)**	5 (100)	5 (100)
**Age, mean (SD)**	30 (8)	30 (8)
**Puncture site**	Abdomen (3), Leg (1), Breast (1)	Axilla (1), Groin (2), Buttock (2)

Table S2: Relative levels of metabolites in skin samples from healthy individuals and patients with Hidradenitis Suppurativa

Table S3. Primers used for qRT-PCR

Gene	Primers
18S	F: CATGGCCGTTCTTAG
	R: CGCTGAGCCAGTCAG
AHR	F: ACATCACCTACGCCAGTCG
	R: CGCTTGGAAGGATTTGACTTGA
AHRR	F: CTTAATGGCTTTGCTCTGGTCG
	R: TGCATTACATCCGTCTGATGGA
CYP1A1	F: AAGGGGCGTTGTGTCTTTGT
	R: ATACACTTCCGCTTGCCCAT
CYP1A2	F: ACAAGGGACACAACGCTGAA
	R: AGGGCTTGTTAATGGCAGTG
HAAO 3	F: AGGACTATCACATCGAAGAGGG
	R: ATGACCACATCCCGGTGTTTC
IDO1	F: GCATTTTTCAGTGTTCTTCGCATA
	R: CATACACCAGACCGTCTGATAGCT
IFN-γ	F: ATTGCAGGCAGGACA
	R: GAGTGTGGAGACCAT
IL-1β	F: CCTGTCCTGCGTGTTGAAAGA
	R: GGGAACTGGGCAGACTCAAA
IL-17A	F: ACTACAACCGATCCACCTCAC
	R: ACTTTGCCTCCCAGATCACA
IL-22	F: TATCACCAACCGCACCTTCA
	R: GTTTCTCCCCAATGAGACGAAC
KAT1, KYAT1	F: CAGACTTTGCCGTGGAAGCCTT
	R: GCACATTCCTGAGCGGGTCTAT
KMO	F: TAGCCCTTTCTCATAGAGGACG
	R: CTCTCATGGGAATACCTTGGGA
KYNU	F: GGGGAAGCGTCCTTGGATTAC
	R: CGTGAAGTTGTAGTTGTGACTCA
QPRT	F: TGGTGAAGGATAACCATGTGGTG
	R: CTGCTGCATTCCACTTCCA
TDO	F: GAACATCTTTTTATCATAACTCATCAAG
	R: ACAACCTTAAGCATGTTCCTTTCAT
TNF-a	F: GAGTGACAAGCCTGTAGCC
	R: GCTGGTTATCTCTCAGCTCCA
TPH1	F: ACGTCGAAAGTATTTTGCGGA
	R: ACGGTTCCCCAGGTCTTAATC
